# Supplementary material for: The Functional Role of Gate Loop Residues in Arrestin Binding to GPCRs
Source: Int J Mol Sci. 2025 Dec 18;26(24):12154. doi: 10.3390/ijms262412154 (PMC12733242; doi:10.3390/ijms262412154)
Supplement: Supplementary file 1 [file ijms-26-12154-s001.zip › ijms-4000177-supplementary.pdf]

Supplemental table S1
Cell-free translation results for gate loop mutants

Arrestin-1 protein	Yield of soluble protein, fmol/μl	% soluble
WT	58.3	82.5
WT	44.5	75.5
Arg291Ala	79.1	79.9
Arg291Glu	114.7	90.5
Gly292Ala	99.8	88.7
ΔGly292	67.8	85.4
Ile293Ala	93.9	88.5
Ala294Leu	71.0	88.3
Leu295Ala	91.0	89.1
Asp296Ala	84.9	81.4
Asp296Arg	65.4	70.3
Gly297Ala	84.8	77.8
ΔGly297	90.5	81.5
Lys298Ala	101.9	89.6
Lys298Glu	96.6	98.2
Ile299Ala	76.6	78.5
Lys300Ala	80.7	80.4
Lys300Glu	101.4	76.1
His301Ala	79.4	85.6
His301Asp	80.8	88.2
Glu302Ala	93.3	92.6
Glu302Arg	66.4	74.7
Asp303Ala	72.4	83.4
Asp303Arg	83.7	77.3
Thr304Ala	82.9	90.7
Asn305Ala	86.8	89.9
Leu306Ala	83.4	77.8
Ala307Leu	76.2	76.9
Arg291Gln	109.3	86.3
Ile293Leu	92.1	88.7
lys298Gln	127	90.6
Ile299Leu	109.9	88.0
Ile293Leu, Ile299Leu	114.7	81.0
His301Gln	100.4	77.3
Glu302Gly	105.7	89.1
His301Gln, Glu302Gly	104.0	85.5

The incorporation of the [^{14}C]leucine into translated protein was determined before and after 1 h centrifugation at 357,000xg (to remove ribosomes and aggregated proteins). The

fraction of soluble arrestin-1 protein (% soluble) is calculated as the ratio of its concentration in the supernatant and in translation mix before centrifugation. Proteins with folding problems give low yields and low % soluble. Note that absolute yields are also affected by the quality of mRNA used (the use of older mRNAs that underwent multiple freeze-thaw cycles results in lower yields). As the mutants were translated in two batches, the data for WT arrestin-1 are shown twice.

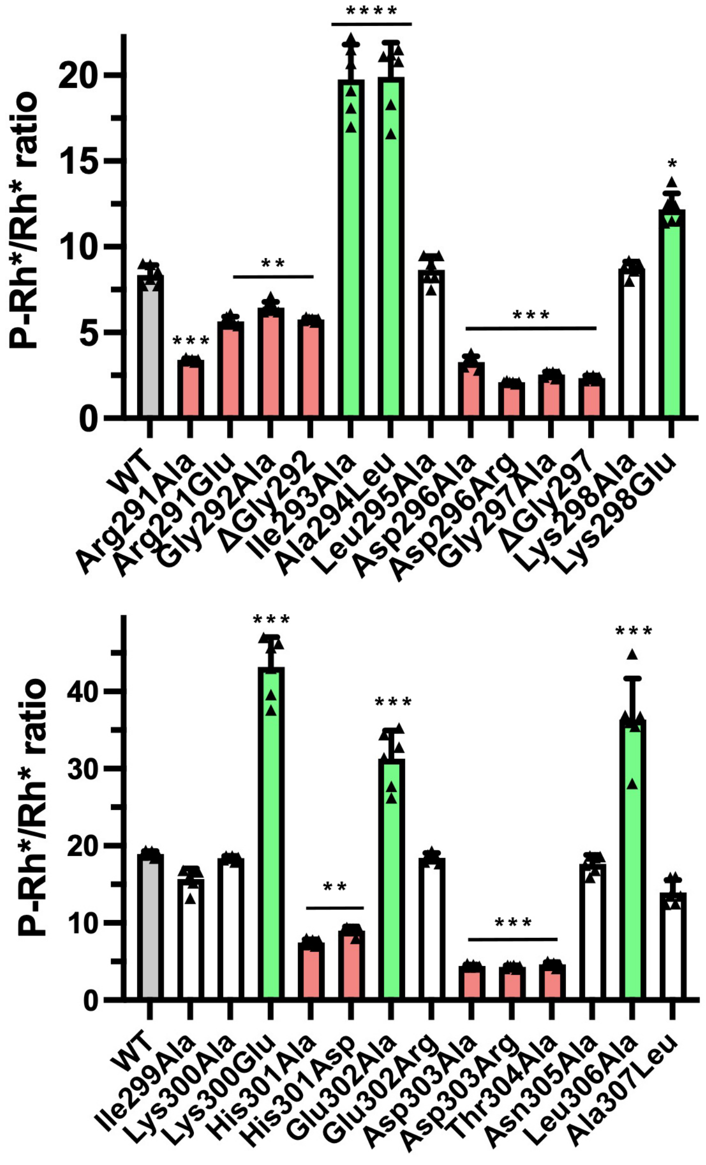

Figure S1. The effect of gate loop mutations on arrestin-1 preference for P-Rh* over Rh*. Calculated from the binding data shown in Fig. 4. Statistical significance of the differences between WT arrestin-1 and mutants was determined by one-way ANOVA followed by Dunnet post hoc comparison to WT with correction for multiple comparisons. Statistical significance (p value) is indicated, as follows: *p < 0.05; **, p<0.01; ***, p<0.001;

****, p<0.0001 to WT. Triangles show the data from individual experiments (n=6). Bars corresponding to mutants with increased or decreased selectivity for P-Rh* are colored light green and light red, respectively; uncolored bars show no significant difference from WT. Bars corresponding to WT are gray. Panels were created by Prism 10 (Graphpad software, Inc., San Diego, CA). The figure was assembled in Adobe Photoshop 2025 (Adobe, San Jose, CA).

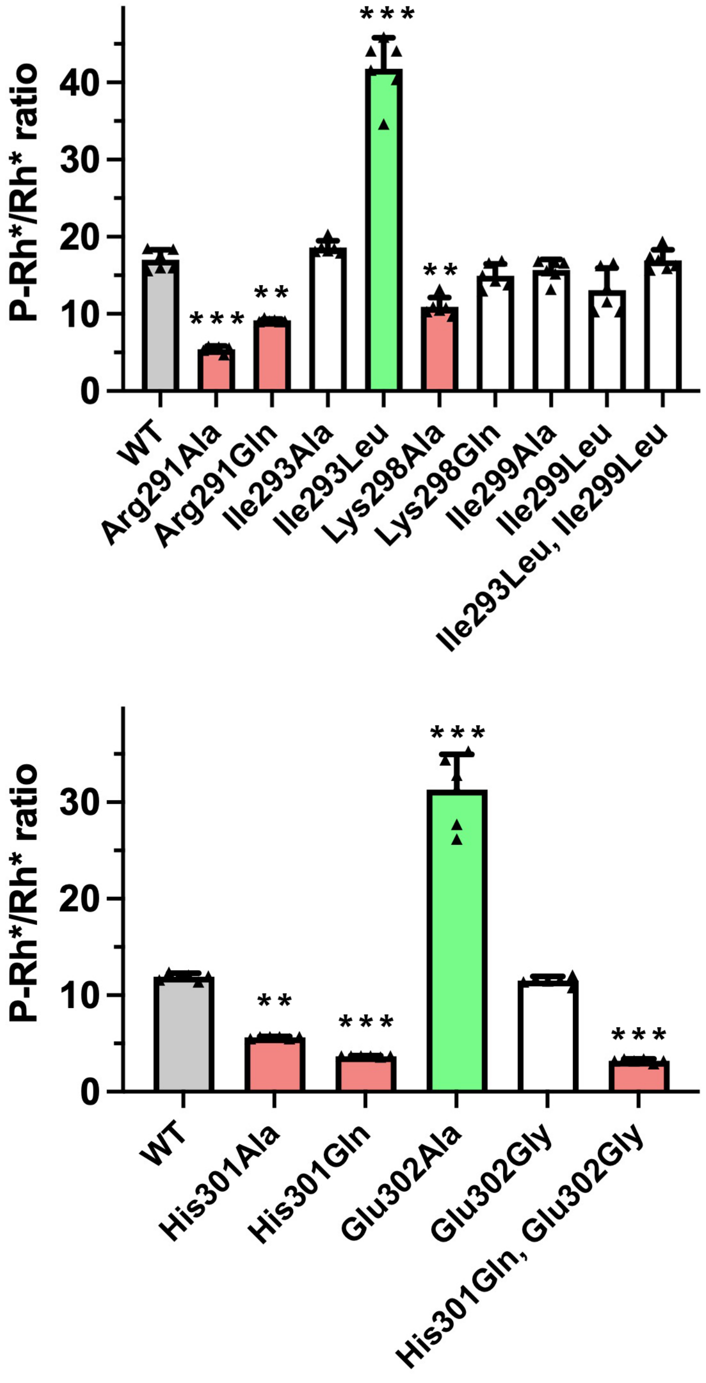

Figure S2. The effect of gate loop mutations mimicking other subtypes on arrestin-1 preference for P-Rh* over Rh*. Calculated from the binding data shown in Fig. 7. Statistical significance of the differences between WT arrestin-1 and mutants was determined by one-way ANOVA followed by Dunnet post hoc comparison to WT with correction for multiple comparisons. Statistical significance (p value) is indicated, as follows: **, $p<0.01$; *** to WT. Triangles show the data from individual experiments (n=6). Bars corresponding to mutants with increased or decreased selectivity for P-Rh* are colored light green and light red, respectively; uncolored bars show no significant difference from WT. Bars corresponding to WT are gray. Panels were created by Prism 10 (Graphpad software, Inc., San Diego, CA). The figure was assembled in Adobe Photoshop 2025 (Adobe, San Jose, CA).
